# Using the Immune System for Effective Periodontal and Caries Management

**DOI:** 10.1155/bmri/6385469

**Published:** 2025-10-31

**Authors:** Salah M. Ibrahim, Ansam Mahdi Khalel

**Affiliations:** ^1^ Department of Oral Surgery, College of Dentistry, Kufa University, Najaf, Iraq, uokufa.edu.iq; ^2^ Department of Oral Diagnosis, College of Dentistry, Kufa University, Najaf, Iraq, uokufa.edu.iq

**Keywords:** adaptive immunity, dental caries, immune response, inflammatory mediators, innate immunity, oral health, oral immunology, periodontal disease, saliva

## Abstract

The immune system plays a critical, albeit complex, role in oral health, mediating the host response to microbial challenges that lead to prevalent diseases like periodontal disease and dental caries. While essential for protection, immune responses, particularly chronic inflammation, can paradoxically contribute significantly to tissue destruction. This comprehensive review uniquely integrates recent advances in oral immunology with clinical applications, providing novel insights into immunomodulatory therapeutic strategies that distinguish it from previous reviews by emphasizing translational approaches and personalized immunogenetic‐based interventions. This review synthesizes current understanding of the intricate interplay between the oral microbiome, innate and adaptive immunity, and the pathogenesis of periodontal disease and caries. We investigate how key immune cells (neutrophils, macrophages, and lymphocytes) and mediators (cytokines and chemokines) orchestrate responses that can lead to alveolar bone loss and enamel demineralization. Saliva′s crucial role in modulating oral immunity is also highlighted. The clinical significance of this work lies in its potential to guide evidence‐based immunomodulatory treatments, improve patient outcomes through personalized therapeutic approaches, and reduce the systemic health burden associated with chronic oral inflammatory diseases. By dissecting these immunological mechanisms, this article is aimed at underscoring the potential for developing novel, targeted immunomodulatory strategies—including vaccines, host modulation therapies, and personalized approaches based on immunogenetics—for more effective prevention and management of these common oral diseases, ultimately promoting better oral and systemic health.

## 1. Introduction

The oral cavity represents a unique immunological environment, hosting a complex ecosystem where the host immune system constantly interacts with a dense and diverse microbial community, the oral microbiome [1, 2]. Maintaining homeostasis in this environment is crucial, yet challenging. Understanding the intricacies of these host–microbe interactions is fundamental to oral health, as disruptions in this delicate balance underpin the etiology and progression of the most common oral diseases: periodontal disease and dental caries.

The specific objectives of this review are to synthesize current knowledge on oral immune mechanisms in periodontal disease and caries pathogenesis, evaluate evidence‐based immunomodulatory therapeutic strategies, identify gaps in current understanding that limit clinical translation, and propose future research directions for personalized oral health management. The scope encompasses both innate and adaptive immune responses, with particular emphasis on translational applications that can inform clinical practice.

Historically, research has predominantly focused on identifying specific putative pathogenic bacteria, such as *Streptococcus mutans*, associated with caries, as well as particular consortia linked to periodontitis [3]. However, a significant paradigm shift has occurred; contemporary understanding emphasizes the critical and often decisive role of the host immune response in disease pathogenesis [4, 5]. Periodontal disease, characterized by inflammation and destruction of tooth‐supporting structures (gingiva, periodontal ligament, and alveolar bone) [6], and dental caries, involving microbial acid–induced tooth demineralization [7, 8], are now recognized as resulting from complex, multifactorial interactions between microbial biofilms and dysregulated or excessive host inflammatory and immune reactions [9, 10].

While microbial factors undoubtedly initiate the process (e.g., plaque accumulation leading to gingivitis [11]), the progression to destructive periodontitis is highly dependent on host factors, giving rise to the concept of the “susceptible host”—individuals predisposed to more severe disease due to their specific immune phenotype, which is influenced by genetic polymorphisms in immune‐related genes (such as IL‐1*β*, TNF‐*α*, IL‐6, and Fc gamma receptor variants [FCGR2A]), epigenetic modifications affecting immune cell function, environmental factors including smoking and stress, and systemic conditions like diabetes that alter immune competency. The immunogenetic foundation of susceptibility involves variations in pattern recognition receptors (PRRs), cytokine production capacity, and regulatory T cell (Treg) function, which collectively determine an individual′s inflammatory response magnitude and resolution capacity [12, 13]. Similarly, while acidogenic bacteria drive the caries process, factors such as salivary composition and flow rate, enamel/dentin structure, and the host′s local immune responses within the saliva and dentin‐pulp complex significantly modulate individual susceptibility and lesion progression [14–16].

**Figure 1:**
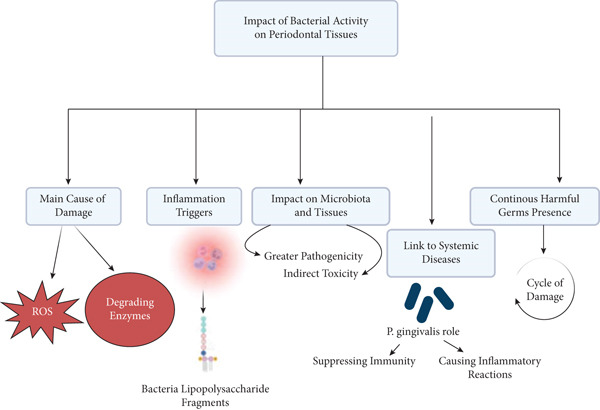
*Porphyromonas gingivalis* and the dysbiotic microbiota in periodontitis. Note: This is an original figure created by the authors.

The immune system, encompassing both the rapid, nonspecific innate arm and the slower, specific adaptive arm, mounts defenses against oral pathogens. However, these very defense mechanisms, particularly chronic inflammation fueled by persistent microbial stimuli and inflammatory mediators, can inadvertently cause significant collateral damage to host tissues [17, 18]. Proinflammatory cytokines, proteases released by neutrophils and macrophages, and osteoclast activation driven by immune cells contribute directly to tissue breakdown [19]. Furthermore, sophisticated pathogens like *Porphyromonas gingivalis* (*P. gingivalis*) actively manipulate host immune responses—for instance, by interfering with complement activation or Toll‐like receptor (TLR) signaling—to evade clearance, promote their survival, and perpetuate chronic inflammation that favors their persistence [20] (Figure 1).

The significance of oral immunity extends beyond the mouth. Robust evidence links chronic oral inflammatory conditions, particularly periodontitis, to increased risk or severity of systemic diseases, including cardiovascular disease, diabetes mellitus, rheumatoid arthritis, adverse pregnancy outcomes, and even neurodegenerative conditions [21–24]. These associations are mediated through multiple pathways: bacteremic dissemination of oral pathogens leading to distant site infections and molecular mimicry, systemic inflammatory spillover via elevated circulating cytokines (TNF‐*α*, IL‐6, and CRP), cross‐reactive immune responses where antibodies against oral bacteria react with host tissues, and shared genetic susceptibility factors that predispose to both oral and systemic inflammatory diseases [25, 26]. Effectively managing oral inflammation is, therefore, crucial not only for preserving oral health but also for contributing to overall systemic well‐being.

The innovative contributions of this review include integrating cutting‐edge immunological research with practical clinical applications, emphasizing personalized therapeutic approaches based on individual immune profiles, providing a comprehensive analysis of emerging immunomodulatory strategies beyond traditional antimicrobial methods, and critically evaluating the translational potential of novel therapeutic targets. This work stands out from existing reviews by offering actionable insights for clinicians while highlighting specific research priorities to advance the field.

This review synthesizes current knowledge on the immunological mechanisms driving periodontal disease and dental caries. By examining the specific roles of innate and adaptive immune cells, signaling molecules, the influence of the oral microbiome, and protective salivary factors, we aim to illuminate how this deepening understanding can be translated into more effective, mechanism‐based, and potentially immunologically targeted strategies for prevention, diagnosis, and therapeutic management in contemporary dental practice.

## 2. The Oral Immune Landscape: Protection and Pathology

The oral immune system operates in a state of controlled inflammation to maintain homeostasis, tolerating the vast commensal microbiota while remaining vigilant against pathogens.

### 2.1. Innate Immunity: The First Line of Defense

Immediate protection relies on physical barriers, soluble factors, and specialized cells [17, 18].

**Figure 2:**
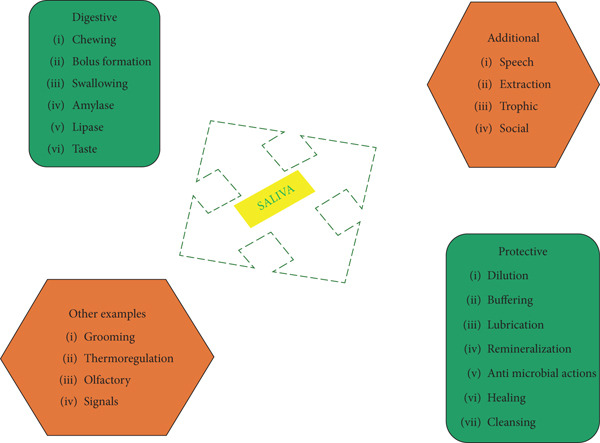
Salivation function. Note: This is an original figure created by the authors.

• Epithelial barrier and salivary defense: Oral epithelial cells form a critical physical barrier while actively participating in immunity by expressing PRRs like TLRs, which detect microbial components (e.g., lipopolysaccharide [LPS] and peptidoglycan), triggering downstream signaling and the production of antimicrobial peptides (AMPs) such as various defensins (*α* and *β*) and cathelicidin (LL‐37) [26]. These AMPs have direct microbicidal activity and immunomodulatory functions. Saliva constantly bathes oral surfaces, providing mechanical cleansing and delivering essential immune molecules, such as lysozyme for bacterial cell wall degradation; lactoferrin for iron sequestration; and the salivary peroxidase system generating hypothiocyanite, histatins, cystatins, and secretory IgA (sIgA) for immune exclusion. Its buffering capacity neutralizes cariogenic acids [27] (Figure 2).
• Phagocytic cells and immune cell interactions: Neutrophils are primary responders to bacterial invasion, rapidly migrating from circulation into tissues (e.g., gingival crevice). They phagocytose bacteria, release granule contents containing proteases (e.g., elastase and MMP‐8) and AMPs, and generate reactive oxygen species (ROS) via respiratory burst [28]. They can form neutrophil extracellular traps (NETs), but excessive NETosis contributes to tissue damage [29]. Macrophages, derived from monocytes, are versatile phagocytes that clear pathogens, cellular debris, and apoptotic neutrophils [28]. Crucially, they serve as antigen‐presenting cells, processing and presenting microbial antigens to T cells, thereby linking innate recognition to adaptive responses. They can polarize into proinflammatory (M1) or anti‐inflammatory/tissue‐repair (M2) phenotypes based on microenvironmental cues [30]. Dendritic cells (DCs) are strategically positioned within the oral mucosa as professional APCs that sample antigens [31]. Upon activation, they mature and migrate to regional lymph nodes, presenting antigens via MHC Class II molecules to naive T cells, establishing the critical bridge between innate sensing and adaptive immunity [32] (Table 1).

**Table 1:** Immune cells and their functions in oral health.

**Cell type**	**Innate/adaptive**	**Location in oral cavity**	**Function**
Neutrophils	Innate	Gingiva and periodontal tissues	Phagocytosis of bacteria and release of antimicrobial substances
Macrophages	Innate	Gingiva, periodontal tissues, and oral mucosa	Phagocytosis, antigen presentation, and cytokine production
Dendritic cells	Innate	Oral mucosa and lymphoid tissues	Antigen presentation and activation of T cells
T lymphocytes (T cells)	Adaptive	Gingiva, periodontal tissues, and lymphoid tissues	Cell‐mediated immunity, cytotoxic activity, and cytokine production

### 2.2. Adaptive Immunity: Specificity and Memory

Adaptive responses provide highly specific, potent, and long‐lasting immunity, involving T and B lymphocytes [32].

• T lymphocytes orchestrate and execute cell‐mediated immunity. Key subsets include the following:
  o. Helper T cells (Th) recognize antigens presented on MHC Class II by APCs. Different subsets produce distinct cytokine profiles: Th1 (IFN‐*γ*) promote cell‐mediated immunity against intracellular pathogens; Th2 (IL‐4, IL‐5, and IL‐13) support humoral immunity and antihelminth responses; and Th17 (IL‐17 and IL‐22) are crucial for mucosal defense against fungi and extracellular bacteria but are strongly implicated in inflammatory tissue destruction (e.g., bone loss) in periodontitis [33]. Follicular helper T cells (Tfh) provide help to B cells in germinal centers.
  o. Cytotoxic T cells (CTLs) recognize antigens on MHC Class I (present on most nucleated cells) and directly kill infected host cells. Tregs express FoxP3 and produce immunosuppressive cytokines, including IL‐10 and TGF‐*β*, to maintain tolerance, suppress excessive immune responses, and prevent autoimmunity. A deficiency or dysfunction of Tregs is implicated in the chronicity of periodontitis [34].
• B lymphocytes are responsible for humoral immunity through the production of antibodies [35]. Upon activation (often requiring T cell help), B cells differentiate into antibody‐secreting plasma cells. In the oral cavity, the dominant antibody isotype is sIgA, produced by plasma cells in salivary glands and lamina propria and transported across epithelial cells [36]. sIgA performs immune exclusion by binding microbes and preventing their attachment to mucosal surfaces. IgG and IgM are also present, particularly in gingival crevicular fluid and inflamed tissues, mediating functions like complement activation and opsonization [37].

**Figure 3:**
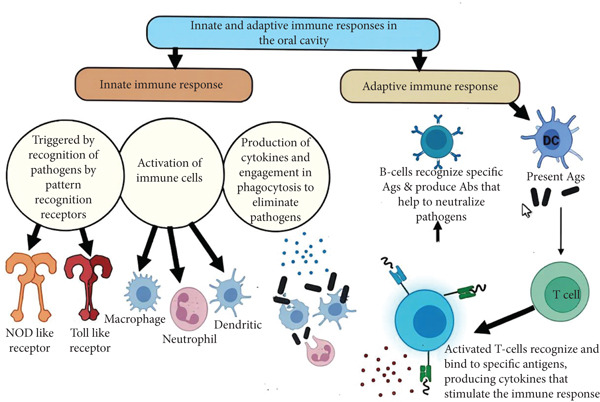
Diagram illustrating the innate and adaptive immune responses in the oral cavity, highlighting the key cells and molecules involved. Note: This is an original figure created by the authors.

Immunological memory is a hallmark of adaptive immunity involving the generation of long‐lived memory T and B cells following initial exposure to an antigen [38]. These cells enable a faster and stronger response upon subsequent encounters with the same pathogen, forming the basis of vaccination (Figure 3). Oral lymphoid tissues include organized lymphoid structures, analogous to gut‐associated lymphoid tissue (GALT), that exist in the oral cavity (e.g., tonsils, adenoids, and salivary gland lymphoid aggregates) and serve as sites for immune induction [39].

### 2.3. The Double‐Edged Sword of Inflammation

Inflammation is a physiological response to injury or infection designed to eliminate the threat and initiate repair. However, if the stimulus persists (like a chronic bacterial biofilm) or the regulatory mechanisms fail, inflammation becomes chronic and destructive [40].

• Mediators: Pro‐inflammatory cytokines, such as TNF‐*α*, IL‐1*β*, and IL‐6, amplify inflammation, increase vascular permeability, recruit immune cells (via chemokines like IL‐8/CXCL8), and induce systemic effects (e.g., fever and acute‐phase response) [41]. IL‐17, primarily from Th17 cells, is a potent driver of neutrophil recruitment and osteoclastogenesis [42].
• Tissue damage: Neutrophils and M1 macrophages release matrix metalloproteinases (MMPs, e.g., MMP‐8 and MMP‐9), cathepsins, and elastase, which degrade extracellular matrix components, such as collagen [43]. ROS contribute to oxidative stress and damage lipids, proteins, and DNA [44]. In periodontitis, cytokines (especially TNF‐*α*, IL‐1*β*, IL‐6, and IL‐17) upregulate the expression of receptor activator of nuclear factor kappa‐B ligand (RANKL) on osteoblasts and T cells, which bind to RANK on osteoclast precursors, driving their differentiation and activation, ultimately leading to alveolar bone resorption [45].
• Failure of resolution: Healthy inflammation ideally concludes with active resolution processes, mediated by specialized proresolving mediators (SPMs), such as lipoxins, resolvins, protectins, and maresins [46]. These SPMs promote neutrophil apoptosis, enhance macrophage efferocytosis (clearance of apoptotic cells), and stimulate tissue repair. Chronic inflammatory diseases, such as periodontitis, may involve impaired SPM production or signaling, leading to persistent, nonresolving inflammation. Understanding these resolution pathways offers therapeutic targets for promoting inflammation resolution rather than merely suppressing inflammatory initiation [47].

## 3. Immunopathogenesis of Major Oral Diseases

The development and progression of both periodontal disease and dental caries are complex processes driven by persistent microbial challenges interacting with intricate host immune and inflammatory responses. While distinct in their primary target tissues (periodontium vs. tooth structure/pulp), both diseases share common themes of microbial dysbiosis triggering host responses that, although intended to be protective, often become pathological and significantly contribute to tissue destruction.

### 3.1. Periodontal Disease: A Dysbiosis‐Driven Inflammatory Condition

Periodontitis is now widely regarded as an inflammatory disease triggered by microbial dysbiosis in susceptible hosts, resulting in the progressive destruction of tooth‐supporting tissues [48, 49]. The process involves distinct stages and a complex interplay of microbial virulence factors, host genetic and environmental modifiers, and a multifaceted immune response.

• Initiation (gingivitis): the initial inflammatory insult

Gingivitis, the reversible precursor to periodontitis, is initiated by the accumulation of dental plaque biofilm at the gingival margin [50]. Bacterial products, particularly LPS from Gram‐negative bacteria and lipoteichoic acid (LTA) from Gram‐positive bacteria, act as pathogen‐associated molecular patterns (PAMPs). These PAMPs are recognized by PRRs, such as TLRs (TLR2 and TLR4), which are expressed on gingival epithelial cells, fibroblasts, and resident immune cells, including macrophages and DCs [51]. This recognition triggers intracellular signaling cascades, such as those involving the MyD88 and NF‐*κ*B pathways, leading to the rapid production and release of proinflammatory cytokines, including TNF‐*α* and IL‐1*β*, as well as chemokines, particularly CXCL8/IL‐8 [52].

• Progression (periodontitis): the transition to chronic destructive inflammation

In susceptible individuals, the initial inflammatory response fails to resolve and transitions into chronic destructive inflammation [50]. Host susceptibility involves genetic factors including polymorphisms in IL‐1*β*, TNFA alleles, and FCGR2A that predispose individuals to dysregulated inflammatory responses [52] (Table 2). Environmental factors such as smoking and poorly controlled diabetes mellitus further exacerbate inflammation through impaired neutrophil function and AGE accumulation [53].

**Table 2:** Immunogenetic factors in oral health.

**Gene name**	**Associated oral condition**	**Mechanism of action**	**Potential implications for personalized dental care**
HLA‐DR2	Periodontitis	Influences immune response to periodontal pathogens and may increase susceptibility	Risk assessment, more frequent monitoring, and targeted preventive measures
IL1B	Periodontitis	Genetic variations can affect the production of IL‐1*β*, a proinflammatory cytokine	Tailored treatment strategies based on individual inflammatory profiles
FCGR2A	Periodontitis	Encodes a receptor for IgG antibodies, variations can influence immune complex clearance	Risk assessment for oral infections, potential for therapeutic use of antimicrobial peptides
DEFB1	Caries and periodontitis	Encodes human *β*‐defensin1, an antimicrobial peptide involved in innate immunity	Risk assessment for oral infections, the potential for therapeutic use of antimicrobial peptides
VDR	Periodontitis and oral cancer	Vitamin D receptor gene, variations can affect immune regulation and cell growth	Potential role of vitamin D supplementation in personalized preventive strategies

Microbial dysbiosis occurs with the enrichment of proteolytic, anaerobic, Gram‐negative bacteria, notably the “red complex” (*P. gingivalis*, *Tannerella forsythia*, and *Treponema denticola*) and other pathobionts such as *Filifactor alocis* and *Aggregatibacter actinomycetemcomitans* [52, 53] (Table 3).

**Table 3:** Oral microbiome composition.

**Bacterial species**	**Associated oral niche**	**Potential benefits/harmful effects**	**Relationship with oral diseases**
*Streptococcus mutans*	Tooth surfaces	Acid production and biofilm formation	Major contributor to dental caries
*Lactobacillus* spp.	Tooth surfaces and mucosa	Fermentation of carbohydrates	Dental caries, potential role in maintaining oral health
*Porphyromonas gingivalis*	Subgingival plaque	Tissue destruction and immune evasion	Key pathogen in periodontitis
*Aggregatibacter actinomycetemcomitans*	Subgingival plaque	Tissue damage and bone resorption	Aggressive periodontitis
*Fusobacterium nucleatum*	Subgingival plaque	Bridge organism in biofilm formation	Periodontitis and oral infections
*Veillonella* spp.	Tongue and mucosa	Nitrate reduction, potential benefits for cardiovascular health	
*Candida albicans*	Mucosa and tongue	Commensal fungus and opportunistic pathogen	Oral candidiasis (thrush) and denture stomatitis
*Streptococcus salivarius*	Tongue and mucosa	Inhibition of other pathogens and potential probiotic	

**Figure 4:**
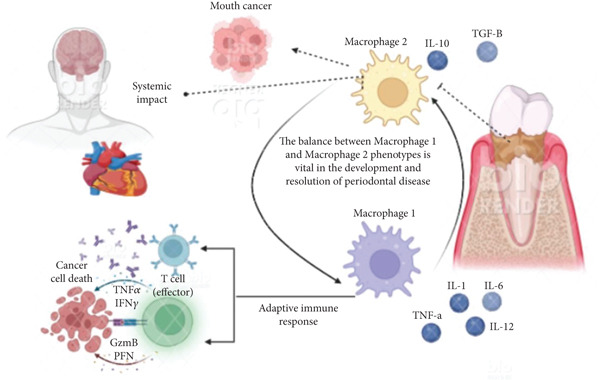
A diagram showing the interaction between the immune system and periodontal pathogens, highlighting the mechanisms of immune evasion and the role of inflammatory mediators. Note: This is an original figure created by the authors.

The “keystone pathogen” hypothesis represents a paradigm shift from traditional “pathogenic bacteria” concepts to understanding microbial dysbiosis. *P. gingivalis* exemplifies this concept through sophisticated immune evasion mechanisms that enable it to manipulate the entire microbial community and host response despite relatively low abundance. Key molecular mechanisms include (1) gingipain proteases that degrade complement components (C3 and C5) and cleave CD14 and TLR4, disrupting innate immune recognition [54]; (2) LPS structural modifications that antagonize TLR4 signaling while promoting TLR2 activation, leading to ineffective immune responses [50]; (3) fimbrial adhesins that interact with complement receptor 3 (CR3) on macrophages, triggering anti‐inflammatory IL‐10 production instead of bacterial clearance [55]; (4) secretion of outer membrane vesicles containing virulence factors that can penetrate host cells and modulate immune responses [56]; and (5) interference with neutrophil function through inhibition of ROS production and promotion of NET formation that damages host tissues [54]. These mechanisms collectively create a dysbiotic environment favoring pathogenic bacteria while suppressing protective immune responses, illustrating the transition from “pathogenic bacteria” to “microbial dysbiosis” perspectives in periodontal disease pathogenesis [55]. This manipulation creates an inflammatory environment (rich in GCF exudate and tissue breakdown products, such as heme and peptides) that favors the growth and persistence of the entire dysbiotic, proteolytic community (Figure 4).

The inflammatory lesion becomes dominated by M1 macrophages, activated T lymphocytes, and plasma cells [57]. Th17 cells producing IL‐17 drive neutrophil recruitment and RANKL expression, linking adaptive immunity to osteoclastogenesis [58]. Treg deficiency contributes to failed resolution [51].

• Tissue destruction

**Figure 5:**
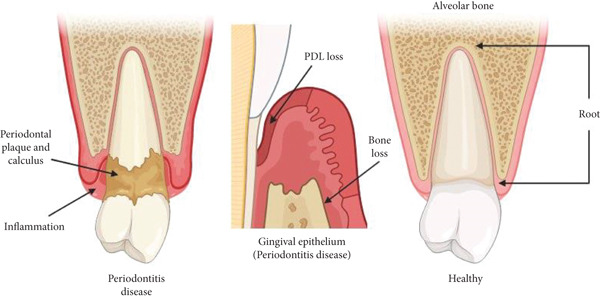
A micrograph showing the structure of periodontal tissues, including gingival epithelium, periodontal ligament, and alveolar bone. Note: This is an original figure created by the authors.

Chronic inflammation mediates periodontal tissue destruction through connective tissue degradation via MMPs (MMP‐8, MMP‐1, MMP‐2, MMP‐9, and MMP‐3) released by neutrophils, macrophages, and fibroblasts, with disrupted MMP:TIMP balance favoring excessive degradation [59]; alveolar bone resorption driven by RANKL/RANK/OPG signaling, where proinflammatory cytokines (IL‐1*β*, TNF‐*α*, IL‐6, IL‐17, and prostaglandin E2 [PGE2]) stimulate RANKL expression and suppress OPG, leading to osteoclast activation and progressive bone loss (Figure 5) [60]; and epithelial alterations including junctional epithelium proliferation and apical migration, forming periodontal pockets that facilitate bacterial product entry and perpetuate inflammation [61].

### 3.2. Dental Caries: Microbial Acid Challenge and Pulpal Immune Responses

Dental caries is fundamentally a disease of localized tooth demineralization caused by acids produced from bacterial fermentation of dietary carbohydrates [62]. Still, the host′s immune system, particularly within the dentin‐pulp complex, plays a significant role in modulating lesion progression and clinical symptoms [63].

• Microbial challenge and biofilm ecology: The process initiates within dental plaque biofilms that adhere to tooth surfaces. Under conditions of frequent exposure to fermentable carbohydrates, mainly sucrose, the biofilm microenvironment becomes acidic [64]. This selects for acidogenic (acid‐producing) and aciduric (acid‐tolerant) bacteria, consistent with the “ecological plaque hypothesis” [65]. Key species include mutans streptococci (*S. mutans* and *Streptococcus sobrinus*), *Lactobacillus* species, *Actinomyces* species, and non‐mutans streptococci [66]. These bacteria rapidly metabolize sugars into organic acids, primarily lactic acid, resulting in a drop in local pH. If the pH falls below a critical level (approximately 5.5 for enamel, slightly higher for dentin) and remains low for a sufficient period, the hydroxyapatite mineral crystals undergo dissolution (demineralization) [67]. The bacteria are embedded within an extracellular polymeric substance (EPS) matrix, primarily composed of glucans synthesized from sucrose by bacterial glucosyltransferases (GTFs). This matrix facilitates bacterial adhesion, consolidates the biofilm structure, and can impede the penetration of buffering saliva and antimicrobial agents while trapping acids near the tooth surface [68].
• Host response: barriers and pulpal defense
  o. Enamel and dentin barriers: Enamel, the outermost layer, is highly mineralized and largely acellular, providing a robust initial barrier. However, it is permeable to acids and bacterial products, especially at microscopic defects or demineralized areas. Dentin, underlying enamel, is a vital tissue containing numerous microscopic tubules that extend from the pulp toward the enamel‐dentin junction (EDJ). These tubules contain odontoblast processes and dentinal fluid, providing pathways for the diffusion of bacterial components, such as acids, enzymes, toxins, and PAMPs like LPS/LTA, from the advancing carious lesion toward the pulp, even before macroscopic pulp exposure occurs [69].
  o. Dentin‐pulp complex immunity: a dynamic defense system: The pulp tissue, rich in blood vessels, nerves, and immune cells, mounts a complex defense response upon sensing microbial threats that diffuse through dentinal tubules [70, 71].
• Odontoblasts as immune sentinels: Odontoblasts, the cells lining the pulp chamber responsible for dentin formation, are now recognized as active participants in innate immunity [72]. They express various PRRs (including TLR2, TLR4, TLR5, TLR9, and NOD receptors) and can directly recognize bacterial PAMPs. Upon activation, they release a range of signaling molecules: chemokines (e.g., CXCL8/IL‐8 and CCL2/MCP‐1) to recruit immune cells like neutrophils and monocytes, proinflammatory cytokines (IL‐1*β*, TNF‐*α*, and IL‐6), and AMPs (*β*‐defensins), and potentially interact with nerve fibers, contributing to neurogenic inflammation via neuropeptides like calcitonin gene‐related peptide (CGRP) and substance P [73]. Odontoblasts also attempt to wall off the insult by depositing tertiary dentin (reactionary dentin if they survive and reparative dentin if they die and are replaced by odontoblast‐like cells) at the pulp–dentin interface beneath the lesion, thickening the dentin barrier [74].
• Pulpal immune cell infiltration and inflammation: As bacterial products reach the pulp, an inflammatory cascade ensues, mirroring events in other tissues but confined within the rigid dentin walls [75]. Neutrophils are rapidly recruited via chemotactic gradients, followed by the infiltration of monocytes that differentiate into macrophages, DCs (which capture antigens for presentation), mast cells (which release histamine and other mediators), and lymphocytes (including T and B cells) [76]. This influx is accompanied by vasodilation, increased vascular permeability, and edema. The subsequent rise in intrapulpal pressure can compromise blood flow and significantly contribute to pain [77].
• Adaptive immune responses in the pulp: Both T and B lymphocytes contribute to the pulpal response. DCs present bacterial antigens to T cells, thereby activating them. Both proinflammatory T cell subsets (Th1 and Th17) and Tregs have been identified in inflamed pulps, suggesting a complex balance determining the inflammatory outcome [78]. B cells differentiate into plasma cells, producing antibodies locally, predominantly IgG, but also IgA and IgM. These antibodies may help neutralize bacterial toxins or opsonize bacteria for phagocytosis; however, excessive immune complex formation could potentially exacerbate inflammation through complement activation [79].
• Immunopathology and clinical outcomes: The pulpal inflammatory response represents a spectrum:
  o. Reversible pulpitis: Characterized by mild to moderate inflammation, often associated with transient pain stimuli, such as cold or sweet stimuli. The pulp retains the capacity to heal if the stimulus (caries) is removed. Immune responses are largely contained and reparative mechanisms (tertiary dentin) are active [80].
  o. Irreversible pulpitis: This condition occurs when inflammation becomes severe and persistent, often due to deeper caries or an overwhelming bacterial challenge [81]. Characterized by spontaneous, lingering, or intense pain. The inflammatory infiltrate is extensive, and microabscesses may form. The elevated intrapulpal pressure can lead to localized ischemia and necrosis. Inflammatory mediators, such as PGE2, bradykinin, histamine, serotonin, and neuropeptides, directly sensitize pulpal nerve fibers, leading to hyperalgesia and spontaneous pain [82]. Cytokines like IL‐1*β* and TNF‐*α* also contribute to pain signaling [83].
  o. Pulpal necrosis: If the inflammation cannot be resolved or contained, widespread cell death occurs, resulting in the necrosis of the pulp tissue. The pulp chamber becomes infected with predominantly anaerobic bacteria [81].
  o. Apical periodontitis: Bacterial products and necrotic debris exiting the root apex trigger inflammation in the periapical tissues, including the periodontal ligament and bone, leading to periapical bone resorption, which is visible radiographically as a radiolucency. This represents a chronic inflammatory lesion containing immune cells similar to those found in periodontitis, which attempts to contain the infection spreading from the root canal system [84].

### 3.3. The Oral Microbiome–Immunity Axis in Disease Context

The bidirectional communication between the oral microbiome and the host immune system is central to the pathogenesis of both periodontitis and caries, albeit through distinct ecological and immunological pathways [85].

**Figure 6:**
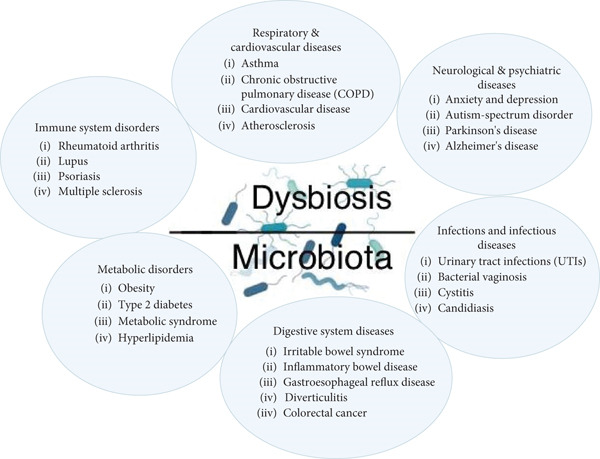
Human microbiota dysbiosis contributes to various diseases. Note: This is an original figure created by the authors.

• Periodontitis context: inflammation‐driven dysbiosis: In periodontitis, inflammation and dysbiosis form a destructive positive feedback loop [86]. Initial inflammation triggered by plaque accumulation alters the local environment, as increased GCF flow provides protein‐rich nutrients (such as heme from bleeding gums and peptides from tissue breakdown) that specifically favor the growth of asaccharolytic, proteolytic pathobionts, like *P. gingivalis* and *T. forsythia* [87]. The inflammatory milieu itself (e.g., altered pH and redox potential) further selects for these organisms. As described earlier, keystone pathogens, such as *P. gingivalis*, actively manipulate host innate immune responses (e.g., complement C5aR/TLR crosstalk) to suppress effective clearance mechanisms while promoting chronic inflammation that benefits the broader dysbiotic community [81]. This sustained dysbiotic challenge then perpetuates the pathological host inflammatory response, driving further tissue destruction, which in turn releases more nutrients, reinforcing the dysbiosis (Figure 6). Host immune status significantly impacts this cycle; for example, conditions like diabetes create a proinflammatory systemic environment that exacerbates local periodontal inflammation and potentially facilitates dysbiosis [88].
• Caries context: environment‐driven dysbiosis and pulpal response: In caries, the primary driver of dysbiosis is environmental change, specifically frequent exposure to dietary sugars, leading to prolonged periods of low pH, as per the ecological plaque hypothesis [89]. This acidic environment selects for acidogenic and acid‐tolerant species, such as *S. mutans* and *Lactobacilli*. While the initial demineralization process is largely physicochemical, the subsequent host immune response is triggered once bacterial products penetrate the dentin barrier and reach the pulp [90]. The composition of the cariogenic biofilm may influence the nature or intensity of the pulpal immune response; for example, different bacterial species produce varying PAMPs or toxins that could elicit distinct odontoblast or immune cell reactions. While the systemic immune status is less directly implicated in caries initiation compared to periodontitis, factors affecting saliva composition and flow (influenced by systemic conditions, medications, or immune disorders such as Sjögren′s syndrome) critically impact the oral environment, thereby influencing microbial ecology and caries risk [91].
• Bidirectional interplay: It is crucial to recognize the constant crosstalk. The host′s innate and adaptive immune systems continuously sample the microbiome, shaping its composition through mechanisms like sIgA‐mediated exclusion or AMP production [36]. Conversely, the microbiome composition profoundly influences the education, maturation, and activation state of both the local and systemic immune systems [92]. Disruptions in either component—whether immune deficiency leading to opportunistic infections or environmental shifts driving microbial dysbiosis, which triggers pathological inflammation–can initiate or exacerbate oral diseases. Understanding this intricate axis is paramount for developing effective prevention and treatment strategies that address both the microbial challenge and the host response [93].

## 4. Leveraging Immunological Knowledge for Management Strategies

The deepening understanding of oral immunopathogenesis provides a strong rationale for developing and implementing strategies that target host responses, complementing traditional antimicrobial and mechanical approaches.

### 4.1. Host Modulation Therapies (HMTs)

HMTs aim to therapeutically modulate the host′s immune and inflammatory responses to reduce destructive processes, primarily explored for periodontitis [94].

• Anti‐inflammatory agents:
  o. NSAIDs: While capable of reducing inflammation, long‐term systemic use has significant side effects. Topical NSAIDs have shown limited, inconsistent benefits [95].
  o. Subantimicrobial dose doxycycline (SDD): The only FDA‐approved HMT for periodontitis, SDD (20 mg bid) works primarily by inhibiting MMPs (collagenase), not by its antibiotic effect, thus reducing collagen breakdown [96]. Clinical evidence from multiple comprehensive systematic reviews and randomized controlled trials demonstrates that SDD as an adjunct to scaling and root planing provides statistically significant improvements in clinical attachment level (0.2–0.4 mm) and probing depth reduction compared to mechanical therapy alone [96, 97]. However, the clinical significance of these modest improvements remains debated, and long‐term safety data regarding prolonged MMP inhibition are limited. Cost‐effectiveness and safety analyses suggest benefits may be marginal for routine use [98].
  o. Targeting specific pathways: Research explores inhibitors of specific kinases (e.g., p38 MAPK and JAK inhibitors) or transcription factors (e.g., NF‐*κ*B) involved in proinflammatory signaling; however, specificity and safety remain significant challenges [99].
• Promoting resolution of inflammation: This innovative approach focuses on enhancing the body′s natural mechanisms to actively terminate inflammation, rather than just blocking proinflammatory mediators [100]. SPMs, such as lipoxins, resolvins, protectins, and maresins, derived from omega‐3 fatty acids, have shown promising results in preclinical studies for SPM application in treating periodontitis [101], with resolvins and lipoxins demonstrating the ability to reduce inflammation and promote tissue regeneration in patients with gingival inflammation, and efficacy results demonstrated significant modified gingival index (MGI) reduction versus no‐rinse control (*p* = 0.041) [101, 102]. A recent Phase II clinical trial of topical resolvin E1 showed modest improvements in gingival inflammation, but larger, well‐controlled trials are needed to establish efficacy and optimal delivery methods [102]. Manufacturing challenges and cost considerations currently limit clinical translation [33].
• Targeting cytokines: Biologics that block key cytokines, such as TNF‐*α* (e.g., infliximab) or IL‐1 (e.g., anakinra), are effective for systemic inflammatory diseases but are generally not viable for routine periodontal treatment due to cost, risk of systemic immunosuppression, and the administration route [79]. Research into locally delivered, targeted cytokine inhibitors or antagonists is ongoing, but it faces challenges related to delivery and retention.

### 4.2. Immunization Strategies

Developing vaccines to prevent caries or periodontitis remains a long‐term goal, though significant hurdles exist [103]. Vaccine development faces substantial immunopathological risks, including potential autoimmune reactions due to molecular mimicry between bacterial and host antigens, the risk of enhancing rather than preventing disease through inappropriate immune responses, and challenges in achieving sustained mucosal immunity [104].

• Caries vaccines: Most strategies target key *S. mutans* virulence factors, such as surface adhesins (AgI/II or PAc) to block attachment, or GTFs to inhibit biofilm matrix formation [104]. The aim is often to elicit a strong, sustained sIgA response in saliva, providing mucosal protection [105]. Delivery routes (e.g., nasal and oral) and adjuvants are critical for inducing effective mucosal immunity [106]. For caries vaccines targeting *S. mutans*, concerns include cross‐reactivity with cardiac myosin and potential disruption of beneficial oral microbiota [107, 108]. A *S. mutans* vaccine showed promise in early trials. Still, it was abandoned due to possible cardiac cross‐reactivity [104, 107]. Ensuring safety, specifically avoiding cross‐reactivity with host tissues, and addressing antigenic diversity among cariogenic strains are significant challenges [109].
• Periodontitis vaccines: Targets often include key virulence factors of *P. gingivalis*, such as fimbriae (FimA), involved in adhesion, or gingipains (proteases crucial for nutrient acquisition and host protein degradation) [110]. Inducing a protective immune response (potentially involving both antibodies and specific T cell subsets) without triggering excessive inflammation is a critical balancing act [79]. Periodontitis vaccines must balance protective immunity against pathogens while avoiding excessive inflammation that could worsen tissue destruction. Effective vaccines require adjuvants to enhance immune responses, but many adjuvants cause excessive inflammation that could damage oral tissues [111]. Vaccines targeting specific bacteria could disrupt beneficial microbiota, potentially leading to dysbiosis and increased susceptibility to other infections [112].

### 4.3. Microbiome Modulation

Interventions aimed at shifting a dysbiotic microbiome back toward a healthier, eubiotic state can indirectly modulate host immunity [113].

• Probiotics: Live microorganisms (often *Lactobacillus* or *Bifidobacterium* strains) that, when administered in adequate amounts, confer health benefits [113]. Clinical evidence for probiotics in oral health shows mixed results. A 2023 systematic review of 25 randomized controlled trials found modest benefits for specific strains (*Limosilactobacillus reuteri* and *Ligilactobacillus salivarius*) in reducing gingival inflammation and plaque accumulation [114, 115]. It found statistically significant improvements in clinical attachment level (weighted mean difference: 0.42 mm) and probing depth reduction (0.35 mm) when used as an adjunct to scaling and root planing [116]. Several studies have evaluated *Lactobacillus casei* formulations with mixed results, showing modest improvements against different types of bacteria [117]. Potential mechanisms in the oral cavity include competitive exclusion of pathogens, production of antimicrobial substances (bacteriocins), and direct modulation of host immune responses (e.g., reducing proinflammatory cytokine production and promoting Treg activity) [118].
• Prebiotics: Nondigestible food ingredients (e.g., certain fibers) that selectively stimulate the growth or activity of beneficial bacteria already present in the host. Their application in oral health is less explored than probiotics [119].
• Targeted bacteriophages or bacteriocins: Utilizing viruses that specifically infect bacteria (phages) or narrow‐spectrum AMPs (bacteriocins) to eliminate specific pathogens while sparing commensal bacteria represents a more targeted future approach [120].

### 4.4. Personalized Dentistry Based on Immunogenetics

Leveraging an individual′s genetic information related to immune responses holds promise for tailoring prevention and treatment [121].

• Risk assessment: Identifying individuals with genetic polymorphisms associated with heightened inflammatory responses (e.g., specific variants in IL‐1*β*, TNFA, FCGR2A, and HLA genes) could enable more intensive preventive measures, such as shorter recall intervals, targeted oral hygiene instructions, or earlier consideration of HMTs [122, 123]. Genetic risk panels are commercially available but require careful interpretation and integration with clinical findings.
• Tailored treatment selection: In the future, immunogenetic profiles may help predict which patients are most likely to benefit from specific HMTs or respond poorly to standard therapies, guiding more personalized treatment planning [124]. Pharmacogenomics could also predict individual responses or adverse reactions to medications used in dental care.
• Ethical and practical considerations: Widespread implementation necessitates addressing issues of cost, accessibility, data privacy, potential for genetic discrimination, and the need for robust clinical validation demonstrating improved outcomes [125].

### 4.5. Addressing Specific Immunological Challenges in Dentistry

Clinical practice often encounters patients with specific immune‐related conditions that require tailored management.

• Allergies: Type I (immediate and IgE‐mediated) and Type IV (delayed and T cell‐mediated) hypersensitivity reactions to dental materials, such as metals like nickel, acrylates in resins, and latex, can occur [126] (Table 4). Thorough medical history taking is crucial. Patch testing may be needed for diagnosis. Management involves identifying and avoiding the allergen, using alternative materials, and treating acute reactions (e.g., antihistamines, epinephrine for anaphylaxis, and corticosteroids for contact dermatitis) [127, 128].
• Immunodeficiencies/immunosuppression: Patients with primary immunodeficiencies (e.g., CGD and LAD) or secondary immunosuppression (due to HIV, chemotherapy, organ transplantation, and immunosuppressive drugs for autoimmune diseases) are at increased risk for opportunistic oral infections (candidiasis, viral infections like HSV/VZV), severe/aggressive periodontitis, poor wound healing, and potentially oral malignancies [129, 130]. Management requires close collaboration with the patient′s physician, meticulous oral hygiene, frequent monitoring, prompt treatment of infections, and potentially prophylactic antimicrobial use [131].
• Autoimmune diseases: Many autoimmune diseases exhibit significant oral manifestations [132, 133]. Examples include Sjögren′s syndrome (dry mouth/eyes due to glandular inflammation and increased caries risk), oral lichen planus (T cell attack on basal keratinocytes causing reticular, erosive, or ulcerative lesions) [134], pemphigus vulgaris (autoantibodies against desmogleins causing intraepithelial blistering/ulceration) [135], mucous membrane pemphigoid (autoantibodies against basement membrane components causing subepithelial blistering), systemic lupus erythematosus (oral ulcers and discoid lesions), and rheumatoid arthritis (TMJ involvement, potential link with periodontitis). Diagnosis often requires biopsy and immunofluorescence. Management typically involves topical or systemic corticosteroids or other immunosuppressants, managed in conjunction with medical specialists [136] (Table 5).
• Oral cancer immunotherapy: Immune checkpoint inhibitors (ICIs) targeting PD‐1/PD‐L1 (e.g., pembrolizumab and nivolumab) have become standard of care for recurrent or metastatic head and neck squamous cell carcinoma (HNSCC), including oral cancers [137]. These drugs activate the patient′s T cells to attack the tumor but can also cause immune‐related adverse events (irAEs), including oral manifestations such as lichenoid reactions, xerostomia, or mucositis [138, 139]. Dentists should be aware of these therapies and their potential oral side effects. CAR‐T cell therapy and therapeutic cancer vaccines are emerging areas [138].

**Table 4:** Allergic reactions to dental materials.

**Type of hypersensitivity**	**Causative agents (examples)**	**Clinical features**	**Treatment options**
Type I (immediate hypersensitivity)	Methacrylates, nickel, latex, and resins	Urticaria, angioedema, and anaphylaxis (rare)	Avoidance of allergens, antihistamines, and epinephrine (for anaphylaxis)
Type IV (delayed hypersensitivity)	Metals (e.g., nickel, chromium, and cobalt), resins, and adhesives	Contact dermatitis, erythema, itching, and oral lichenoid reactions	Avoidance of allergens, topical corticosteroids, and immunomodulatory agents
Type I/IV (mixed hypersensitivity)	Methacrylates, resins, and composites	Combination of immediate and delayed reactions, including urticaria, itching, and dermatitis	Avoidance of allergen, combined treatment approaches for Type I and Type IV reactions

**Table 5:** Immunological aspects of oral diseases.

**Oral disease**	**Key immunological features**	**Role of specific immune cells/molecules**	**Potential therapeutic targets**
Periodontal disease	Chronic inflammation, dysregulated immune response, and tissue destruction	Neutrophils, macrophages, T cells, and cytokines (IL‐1 and TNF‐*α*)	Anti‐inflammatory agents, cytokine inhibitors, and modulation of specific immune cell subsets
Dental caries	Dysbiosis of oral microbiome and inflammatory response to cariogenic bacteria	Neutrophils, B cells (IgA production), and antimicrobial peptides	Vaccines against cariogenic bacteria and modulation of oral microbiome
Oral lichen planus	T cell‐mediated autoimmune response and chronic inflammation	Cytotoxic T cells and cytokines (IFN‐*γ* and TNF‐*α*)	Immunosuppressive agents and T cell targeted therapies
Pemphigus vulgaris	Autoantibodies against desmoglein proteins and blister formation	B cells (autoantibody production) and complement system	Immunosuppressive agents and B cell depletion therapy
Recurrent aphthous stomatitis	Complex immune dysregulation, T cell involvement, and inflammatory mediators	T cells and cytokines (IL‐1*β* and TNF‐*α*)	Topical corticosteroids and immunomodulatory agents
Oral candidiasis	Opportunistic fungal infection and impaired immune response	Neutrophils, macrophages, T cells, and antifungal peptides	Antifungal agents and modulation of immune response

### 4.6. Clinical Implementation Framework and Treatment Priorities

Clear guidance on treatment priorities. Implementing immunologically informed strategies requires systematic risk assessment and evidence‐based decision‐making. We suggest a three‐step approach that balances effectiveness, safety, and cost considerations.

• Evidence‐based standard care (immediate implementation)
o. Risk stratification: a comprehensive assessment including genetic risk factors (IL‐1 polymorphisms), salivary immune function—particularly sIgA production—and AMP activity [140], along with clinical parameters (bleeding on probing and clinical attachment loss) and systemic factors (diabetes and smoking) [141].
  o. Enhanced conventional therapy: scaling and root planing with adjunctive antimicrobial therapy for high‐risk patients, emphasizing thorough biofilm disruption and patient education [142]. Restorative materials should be selected based on their biocompatibility and effects on local immune responses [143].
  o. Proven host modulators: use SDD selectively for patients with aggressive disease patterns or poor response to conventional therapy after a careful risk‐benefit assessment [144]. Fluoride therapy should be tailored based on individual immune status and caries risk, considering the immune‐modulating effects of different fluoride formulations [145]. Casein phosphopeptide–amorphous calcium phosphate (CPP‐ACP) and other remineralizing agents may offer additional benefits for immune‐compromised patients [146].
• Targeted immunotherapy (selective implementation)
  o. Patient selection: high‐risk patients with genetic susceptibility markers, systemic inflammatory diseases, or refractory periodontitis despite optimal conventional therapy [147].
  o. Emerging therapies: carefully monitored use of SPMs, targeted probiotics, or anti‐inflammatory agents in clinical trial settings or compassionate use protocols [148]. Patients with compromised immune systems require modified caries management protocols that account for reduced immune responses and increased infection risk [149].
  o. Biomarker monitoring: regular assessment of inflammatory markers (IL‐1*β*, MMP‐8, and C‐reactive protein) to guide treatment decisions and monitor response [150].
• Experimental approaches (research settings)
  o. Clinical trial participation: consideration of experimental therapies, including vaccines, cell‐based treatments, or novel immunomodulators, for patients with severe, refractory disease [151].
  o. Personalized medicine protocols: integration of genetic testing, microbiome analysis, and immune profiling to guide individualized treatment strategies [152].
  o. Outcome assessment: comprehensive monitoring including clinical, microbiological, immunological, and patient‐reported outcomes [153].

## 5. Conclusion

The understanding of periodontal disease and dental caries has undergone a profound evolution, shifting beyond a purely infectious disease model to encompass the critical role of the host immune response in determining clinical outcomes. Oral immunity is recognized as a complex and dynamic system, where defense mechanisms, although essential, can become dysregulated and drive significant pathology when faced with chronic microbial challenges or specific host susceptibilities. The intricate interplay between the diverse oral microbiome, protective salivary components, and the multifaceted innate and adaptive immune pathways governs the delicate balance between oral health and disease.

This synthesis underscores that the future of effective prevention and management for these ubiquitous oral diseases lies increasingly in strategies that intelligently modulate the host response, alongside traditional approaches.

Despite significant advances, several limitations constrain clinical translation of immunological knowledge. The complexity of oral immune responses makes it challenging to predict individual patient responses to immunomodulatory therapies. Most clinical evidence for novel therapeutic approaches comes from small, short‐term studies with limited follow‐up. The heterogeneity of periodontal and caries disease presentations complicates the development of standardized treatment protocols. Cost‐effectiveness data for emerging therapies are limited, which may limit accessibility. Regulatory pathways for immunomodulatory oral health products remain complex and time‐consuming. Finally, the translation gap between promising preclinical results and clinical efficacy remains substantial.

Future research directions with priority ranking: HIGH PRIORITY—developing validated biomarkers for personalized risk assessment and treatment monitoring, supported by advancing genomic and proteomic technologies. HIGH PRIORITY—conducting large‐scale, long‐term clinical trials of promising immunomodulatory therapies, which require significant investment but are essential for evidence‐based practice. MEDIUM PRIORITY—integrating artificial intelligence and machine learning for personalized treatment planning, feasible with current technology but needing extensive validation. MEDIUM PRIORITY—developing targeted drug delivery systems for oral immunomodulatory agents, technically challenging but promising based on advancements in other medical fields. LOWER PRIORITY—applying single‐cell RNA sequencing in oral health, which, while scientifically interesting, faces major technical and cost barriers for routine clinical use. Research funding should prioritize high‐impact, clinically translatable studies over purely mechanistic research.

This review establishes oral health as a critical determinant of systemic health and positions oral‐systemic health integration as a fundamental component of modern healthcare delivery. The robust evidence linking periodontal disease to cardiovascular disease, diabetes, adverse pregnancy outcomes, and other systemic conditions demonstrates that oral health cannot be considered in isolation from overall health. The bidirectional nature of these relationships, where systemic conditions influence oral health and oral diseases impact systemic health, necessitates integrated approaches that address both local and systemic factors simultaneously. Healthcare systems that fail to recognize and address these connections miss critical opportunities for chronic disease prevention, early intervention, and improved patient outcomes while potentially increasing healthcare costs through fragmented care delivery. The integration of oral health into systemic health management offers unprecedented opportunities for improving population health outcomes. Periodontal therapy in diabetic patients can achieve clinically significant improvements in glycemic control comparable to some antidiabetic medications, potentially reducing diabetes complications and healthcare costs. Cardiovascular risk reduction through oral health management represents a novel preventive strategy that could complement traditional risk factor modification. Pregnancy outcomes may be improved through maternal oral health interventions, potentially reducing preterm birth rates and associated neonatal complications. The emerging understanding of oral–brain connections suggests potential for oral health interventions in neurodegenerative disease prevention. These opportunities position oral health professionals as key contributors to preventive medicine and chronic disease management, expanding their role beyond traditional oral health boundaries to encompass systemic health promotion.

## Disclosure

The funders were not involved in the study design, data collection, data interpretation, manuscript writing, or the decision to submit the paper for publication.

## Conflicts of Interest

The authors declare no conflicts of interest.

## Author Contributions

Salah M. Ibrahim: conceptualization, literature search and data synthesis, writing—original draft, writing—review and editing, visualization, and supervision. Ansam Mahdi Khalel: literature search and data synthesis, writing—original draft, writing—review and editing, and visualization.

## Funding

This research received no external funding. The authors declare that no funds, grants, or other support were received during the preparation of this manuscript.
